# Correlation detection as a general mechanism for multisensory integration

**DOI:** 10.1038/ncomms11543

**Published:** 2016-06-06

**Authors:** Cesare V. Parise, Marc O. Ernst

**Affiliations:** 1 Cognitive Neuroscience Department and Cognitive Interaction Technology-Center of Excellence, Bielefeld University, 33615 Bielefeld, Germany; 2 Max Planck Institute for Biological Cybernetics, 72076 Tübingen, Germany; 3 Applied Cognitive Psychology, Faculty for Computer Science, Engineering, and Psychology, Ulm University, 33615 Ulm, Germany

## Abstract

The brain efficiently processes multisensory information by selectively combining related signals across the continuous stream of multisensory inputs. To do so, it needs to detect correlation, lag and synchrony across the senses; optimally integrate related information; and dynamically adapt to spatiotemporal conflicts across the senses. Here we show that all these aspects of multisensory perception can be jointly explained by postulating an elementary processing unit akin to the Hassenstein–Reichardt detector—a model originally developed for visual motion perception. This unit, termed the multisensory correlation detector (MCD), integrates related multisensory signals through a set of temporal filters followed by linear combination. Our model can tightly replicate human perception as measured in a series of empirical studies, both novel and previously published. MCDs provide a unified general theory of multisensory processing, which simultaneously explains a wide spectrum of phenomena with a simple, yet physiologically plausible model.

## Abstract (web summary)

The human brain integrates inputs across multiple sensory streams into a unified percept. Here Parise and Ernst present a model that assesses the correlation, lag and synchrony of multisensory stimuli, and predicts psychophysical performance on multisensory temporal and spatial tasks.

## Graphical abstract

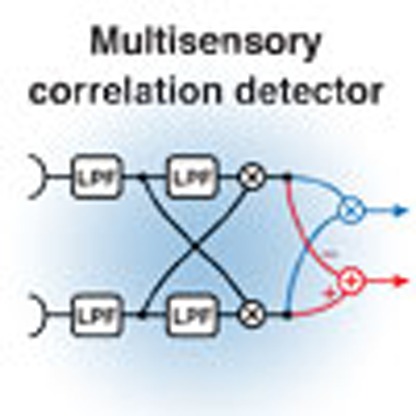

One of the most fundamental aspects of the brain is to effectively process multisensory information. All animals—even the simplest ones–are equipped with multiple sensory organs to perceive and interact with their surroundings. To successfully combine signals from different sensory modalities, the brain needs to detect which signals contain related information, that is, solve the correspondence problem, integrate this information and dynamically adapt to spatial or temporal conflicts across the senses as they arise^1^,^2^. Spatiotemporal correlation has often been advocated as the main common factor underlying the sensory signals to be integrated^3^,^4^,^5^,^6^,^7^,^8^: when signals from different modalities originate from the same physical event, and hence contain related information that should be integrated, they usually cross-correlate in time and space. Sensory neuroscience has already acknowledged the fundamental role of correlation detection in multisensory processing^5^,^6^, and recent studies have demonstrated that multisensory cue integration is statistically optimal only when signals are temporally correlated^3^,^4^, although this effect seems to disappear at high temporal frequencies^9^. To date, there is no single model that can provide a unified explanation for the manifold aspects of early multisensory processing: how does the brain process multisensory signals to detect correlation and temporal lags across the senses? How does it solve the multisensory correspondence problem? And how does it eventually achieve optimal cue integration of redundant signals?

Correlation detection is at the core of any computational principle for combining different sensory signals, and it is widely exploited throughout the animal kingdom. This is the case for binaural hearing^10^, binocular vision^11^ and visual motion perception^12^, to name just a few. In motion perception, for example, within the continuous stream of visual inputs, the brain needs to compare the luminance of two neighbouring receptive fields over time to detect speed and direction of motion. On the basis of the insect oculomotor reflex, Hassenstein and Reichardt^12^ proposed a biologically plausible cross-correlation model for motion perception. This model, known as the Hassenstein–Reichardt detector (or elementary motion detector), posits the existence of two mirror-symmetric subunits. In its simplest version, each subunit multiplies inputs from two neighbouring visual receptive fields after applying a delay (or low-pass temporal filtering) to one of those signals. The difference between the outputs of these subunits eventually determines the perception of motion and its direction. Over more than five decades, this model has been successfully applied to explain motion perception also in vertebrates and humans^13^, and neurophysiological studies support the existence of such a mechanism in the insect visual system, such as the fly optic lobe^14^. To date, the Hassenstein–Reichardt detector is possibly the neural model whose biological substrates are best understood, and whose computational steps have been recently identified even at the level of individual cells^15^,^16^.

The basic architecture of the Hassenstein–Reichardt detector displays a number of important properties that would also be useful for multisensory processing. For example, if a multisensory processing unit akin to the Hassenstein–Reichardt detector would receive inputs from different modalities, it could compute the cross-correlation across the senses, and hence solve the correspondence problem^3^,^4^. Moreover, Hassenstein–Reichardt detectors are naturally suited to detect the relative time of arrival of two separate signals: the very same mechanism that in visual perception detects the direction of motion could also be used crossmodally to detect temporal lags across the senses. Here we show that a neural mechanism similar to an elementary motion detector can concurrently explain several aspects of multisensory processing, including the detection of simultaneity, correlation and lag across the senses, and Bayesian-optimal multisensory integration.

## Results

### Model and psychophysical experiment

**Figure 1:**
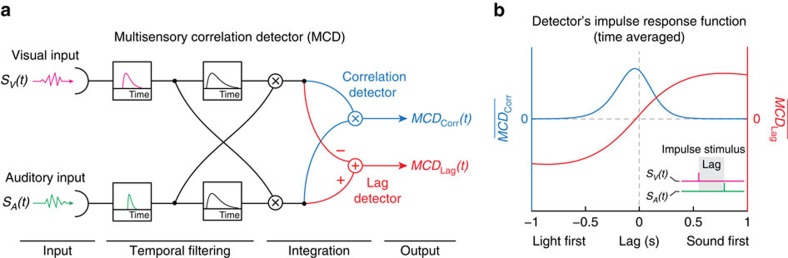
MCD model. (**a**) Schematic representation of the model. The MCD integrates multisensory signals (*S*_V_(*t*), *S*_A_(*t*)) through a set of low-pass temporal filters followed by linear operations. The MCD model yields two outputs, *MCD*_Corr_(*t*) (equation 4) and *MCD*_Lag_(*t*) (equation 5), representing, respectively, the temporal correlation and lag across the input signals. (**b**) Time-averaged impulse response function of the MCD. The *y* axis represents the response of the model to visual and auditory impulses as a function of the lag across the senses (see inset). Blue line and axis represent the time-averaged response of the correlation detector (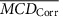, equation 6), red line and axis represent the time-averaged response of the lag detector (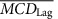, equation 7). Note how the correlation detector output (blue) peaks at low lags, whereas the output of the lag detector (red) changes sign depending on which modality comes first.

The structure of the multisensory correlation detector (MCD, see Methods) proposed here closely resembles the Hassenstein–Reichardt detector that was originally developed to explain visual motion perception. However, instead of receiving visual information from neighbouring receptive fields, the MCD receives inputs from spatially aligned receptive fields of different senses. In a first processing stage, multisensory input signals undergo separate low-pass temporal filtering (Fig. 1a, magenta and green filters). This accounts for the impulse response characteristics of each individual sense during transduction, transmission and early unisensory processing^6^. These filtered signals are then fed into two mirror-symmetric subunits, which multiply the signals after introducing a temporal shift to one of them through another low-pass filter (Fig. 1a, centre, black filters). As a consequence of this additional filtering stage, each subunit is selectively tuned to different temporal order of the signals (that is, vision vs. audition lead). The outputs of the two subunits are then combined in different ways to detect correlation and lag of multisensory signals, respectively (Fig. 1a, right). Specifically, correlation is calculated by multiplying the outputs of the subunits (Fig. 1a, top-right; equations 4, 5, 6; Supplementary Video 1; and Supplementary Fig. 1A), hence producing an output (*MCD*_Corr_) whose magnitude represents the correlation between the signals (Fig. 1b, blue lines). Temporal lag is instead detected by subtracting the outputs of the subunits, like in the classic Hassenstein–Reichardt detector (equations 5, 6, 7, Supplementary Video 1 and Supplementary Fig. 1B). This yields an output (*MCD*_Lag_) with a sign that represents the temporal order of the signals (Fig. 1b, red lines).

**Figure 2:**
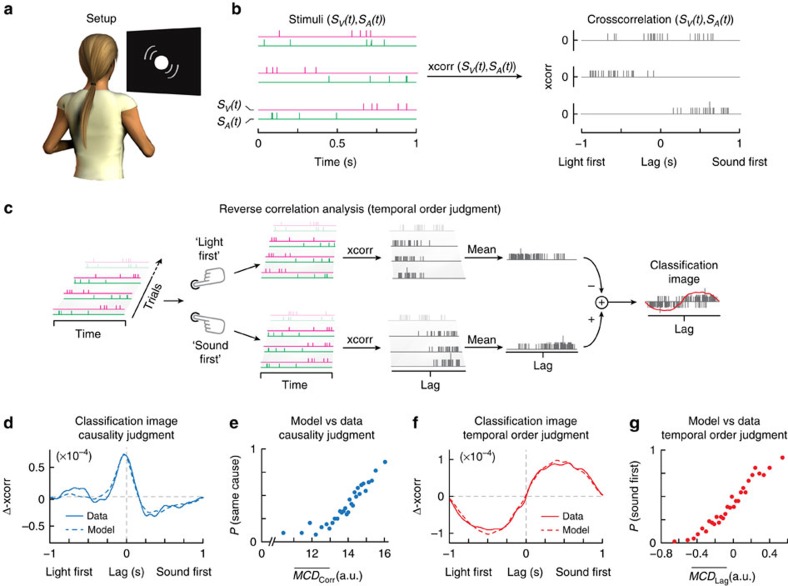
Stimuli, reverse-correlation analyses and results of the psychophysical experiment. (**a**) Experimental setup. Participants sat in front of a white fabric disc covering an LED and a speaker. (**b**) Examples of stimuli used in the experiment (left side), and their cross-correlation (right). Magenta and green lines represent visual (*S*_V_(*t*)) and auditory stimuli (*S*_A_(*t*)), respectively. The top row shows an audiovisual stimulus eliciting high 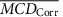 responses; the lower two elicit low and high 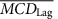 responses, respectively. Cross-correlation of the first stimulus is high at short lags; in the other two it is higher at negative and positive lags, respectively. (**c**) Reverse-correlation analyses. Stimuli were classified according to participants' responses, that is, ‘light' vs. ‘sound first' in the temporal order judgment task (or ‘same' vs. ‘different causes' in the causality judgment task, not shown). Classification images were calculated by subtracting the average cross-correlation of trials classified as ‘sound first' from the average cross-correlation of trials classified as ‘light first', and smoothing the results using a Gaussian kernel (*σ*=20 ms, red line, see also **f**). (**d**,**f**) Classification images (solid lines represent data, dashed lines the model). Positive values on the *y* axis represent positive association to ‘same cause' or ‘sound-first' responses. Predicted classification images are vertically scaled. (**e**,**g**) Model output (equations 6, 7) plotted against human responses. Each dot corresponds to 315 responses, 63 per participant. See Supplementary Fig. 2 for plots of individual observers' data. LED, light-emitting diode.

Without losing generality, we here focus on the integration of time-varying signals from vision and audition. To probe multisensory correlation detection in humans, and hence to test the MCD model, five human observers performed a psychophysical forced-choice task (Methods). On each trial we presented a complex sequence of five auditory and five visual impulses (that is, sequences of clicks and flashes) with random temporal structures (Fig. 2a,b and Supplementary Video 2). Participants had to report both whether the visual and auditory sequences appeared to share a common cause (causality judgment), and which of the two sequences—vision or audition—came first (temporal order judgments). The temporal structures of the visual and auditory signals were generated independently, and varied randomly across trials (*n*=1890). Such stimuli were selected because they emphasize the role of cross-correlation for solving the correspondence problem, while the experimental tasks were selected because they directly probe the detection of multisensory correlation (causality judgment) and lag (order judgment). The stochastic nature of the signals implies the lack of a ground truth on which to devise an optimal classifier. Specifically, given that there were five randomly placed impulses per modality on each trial, there was no univocal way to decide about the relative temporal order or about the common causal structure of the signals, hence rendering the task fundamentally subjective.

Human responses were analysed using psychophysical reverse-correlation techniques (Methods). By measuring how random variations in the visual and auditory stimuli correlate with participants' responses, this technique allows discriminating which properties of the audiovisual signals (for example, correlation, lag, temporal filtering and so on) selectively determine human perception (that is judging order or causality). Compared with classical psychophysical techniques, reverse-correlation analyses offer a more stringent test for the MCD model, as they allow assessing—without explicit experimental manipulations—whether humans and MCD base their responses on the same stimulus dimensions.

Given that both causality and temporal order judgments rely on the joint temporal properties of visual and auditory stimuli, reverse-correlation analyses were performed on the cross-correlation profile of the signals. Cross-correlation provides a measure of similarity of the signals as a function of lag, and it highlights common features across complex signals. The classification image for causality judgments demonstrates—as might be intuitively predicted—that signals with high correlation at short lags are more likely perceived as sharing a common cause^3^,^4^,^17^ (Fig. 2d). Notably though, it displays a negative lobe on the sound-first side, indicating that the brain has a tendency not to integrate audiovisual information when sound arrives first, a tendency that mirrors natural signal statistics given that light travels faster than sound. Conversely, as expected, the responses in the temporal order judgments were driven by the sign of the lag at maximum cross-correlation (that is, light vs. sound lead; Fig. 2f). More generally, these results demonstrate that the underlying neural processes are sensitive to the correlation of the signals; otherwise no clear pattern would have emerged in the classification images calculated from the cross-correlation.

Having determined the precise shape of the empirical classification images, we can now assess how closely the MCD matches human performance. To do so, we feed the same signals used in the experiment into the model, and perform reverse-correlation analyses on the model responses just as we did with human responses (see Methods). This way, we can determine the temporal constants of the model's low-pass filters by fitting them to maximize the similarity between empirical and predicted classification images (three free parameters, see Methods). As can be seen in Fig. 2d,f, this produces an excellent agreement between human data and model responses, which demonstrates that the MCD accurately captures some fundamental aspects of the neural computation underlying human perception. The model can near-perfectly reproduce the shapes of both empirical classification images (Fig. 2d,f): Pearson's correlation between empirical and predicted classification images is *ρ=*0.97 for the causality judgment, and *ρ=*0.99 for the temporal order judgment, respectively (see Supplementary Fig. 2A,C for individual participants' results). Because of the different temporal constants of vision and audition, the model could also reproduce the same negative lobe in the classification image of the causality judgment on the sound-first side. Note that due to additional noise in the neural processing, the empirical classification images were shallower than those predicted by the model. This additional noise was taken into account by scaling the classification images produced by the MCD, thereby highlighting the similarity between the simulation and the empirical findings.

To further test the predictive power of the MCD—besides reverse-correlation analyses—we investigated whether, using the same fitted parameters determined from the classification images, the model (equations 6 and 7) could predict human responses when given the same stimuli as input (Methods). For both tasks we found a strong monotonic mapping between predicted and empirical responses (Fig. 2e,g; Spearman's rank correlation between model output and human responses: *ρ=*0.96 for causality judgments; *ρ=*0.99 for temporal order judgments; see Supplementary Fig. 2B–D for individual participants results).

Previous attempts to model multisensory temporal perception lack the flexibility to concurrently deal with the tasks and the complex signal streams used here. For example, both Sternberg and Knoll^18^ and Cai *et al*.^19^ proposed models that describe human performance in temporal order judgment tasks given the delay between two signals as input. However, it has never been explained how such delays are detected in the first place, particularly in the presence of complex time-varying signal streams (as they occur in the natural world).

Burr *et al*.^6^ proposed a model for audiovisual duration discrimination. Although this model can also be used to process the current stimuli, it cannot account for the current results (see Supplementary Note 1). Regarding correlation (but not lag) detection, Fujisaki and Nishida^5^ proposed a rudimentary descriptive scheme, that hold some similarities to the MCD model, in form of a correlator between the visual and auditory signals (see Supplementary Note 2). However, the working principle of this correlation detector has not been formally specified. In contrast, the MCD model makes all such key computational steps explicit, is flexible enough to provide quantitative predictions for both causality and temporal order judgments, and can meaningfully process stimuli of any complexity, a property that is inevitable for handling real-world situations.

### Validation of the MCD through simulation of previous results

Given that there is an extensive literature on multisensory perception of correlation, simultaneity, and lag^5^,^17^,^20^, we can validate the MCD and assess its generalizability by comparing its responses against human performance as determined in earlier experiments. To this end, we selected a series of studies that employed parametric manipulations of the temporal structure of the signals, we simulated the stimuli, and we used the MCD model (with fixed filter parameters) to predict human performance. That is, it is important to stress that in the following predictions we used the temporal constants of the MCD that we determined in the previous experiment, such that now there are no free parameters of the MCD to fit. The output of the model was related to a response probability by assuming that the model response is corrupted by late noise, and that perceptual judgments are based on a decision criterion. As standard practice in psychophysics, we modelled this stage using a general linear model (GLM) with two free parameters (that is, noise and criterion). If the MCD is the basic computational unit for multisensory temporal processing, we should be able to reproduce all of the earlier findings on the perception of multisensory temporal attributes with this constrained MCD.

### Audiovisual correspondence detection

**Figure 3:**
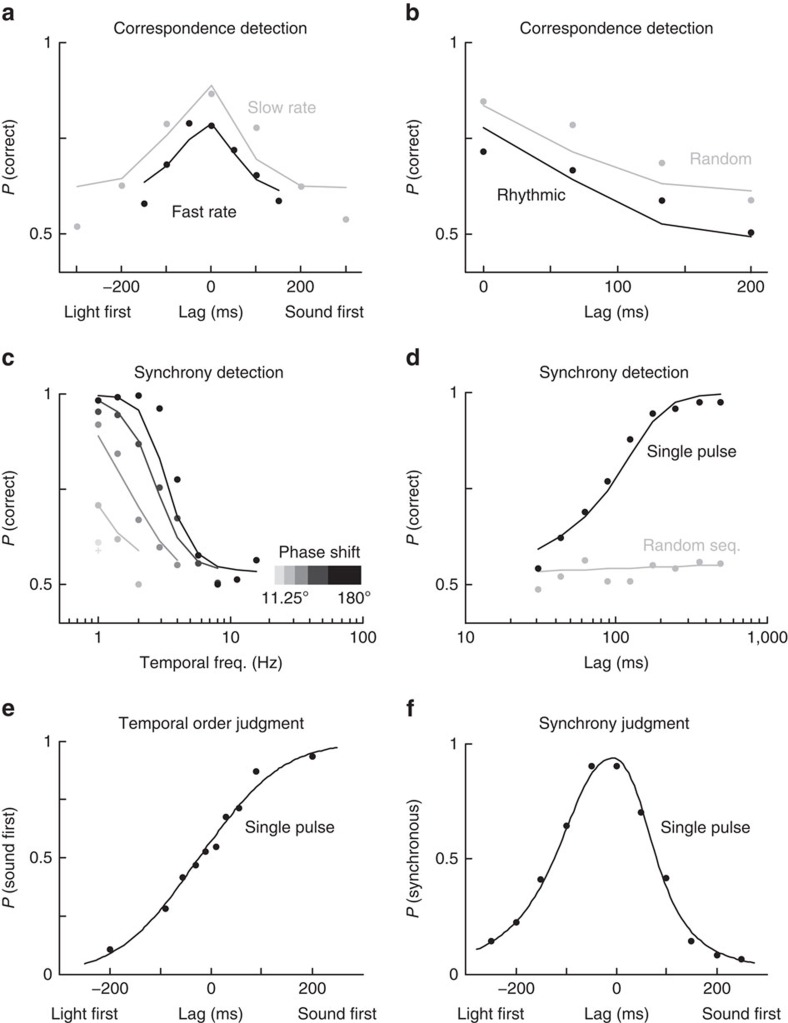
Comparison of our model to previously published psychophysical results. Dots represent the empirical data, lines the model prediction. (**a**) Effects of lag and stimulus rate in a correspondence detection task (data from ref. 17, Experiment 1). (**b**) Effects of lag and stimulus rate on correspondence detection (data from ref. 17, Experiment 3). (**c**) Effects of temporal frequency (rate) and phase shift of periodic stimuli on synchrony detection (data from ref. 5, Experiment 1). (**d**) Synchrony detection for a single pulse (data from ref. 5, Experiment 1) and random sequences of pulses (temporal rate 80 Hz, data from ref. 20, Experiment 1). (**e**) Temporal order judgment task (data from ref. 21, Experiment 2). (**f**) Synchrony judgment task (data from ref. 23, Experiment 1).

To measure the determinants of multisensory correspondence detection, Denison *et al*.^17^ presented streams of audiovisual events with random temporal structures that were sometimes correlated, and they systematically varied lag, rhythmicity and rate. In a forced-choice task, participants had to detect audiovisual correspondence (Methods). That is, participants had to report which of two visual stimuli had the same temporal structure as the auditory stimulus. Their results demonstrate that correspondence detection systematically depended on the temporal properties of the signals and their complexity: performance decreased with lag, rhythmicity and rate (Fig. 3a,b, dots, see ref. 17, Experiments 1 and 3). Feeding these stimuli to the model demonstrates that, in line with human behaviour, 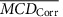 (equation 6) responses also decrease with lag, rhythmicity and rate, effectively replicating all the patterns in the original data set without the need to adjust any of the time constants (Fig. 3a,b, lines; see Methods and Supplementary Table 1 for details).

This result may also explain the previously reported null effects of correlation on multisensory integration of audiovisual stimuli at high temporal rates (between 7 and 15 Hz, see ref. 9). The reason may be that due to its low temporal resolution as a result of the low-pass filtering, the human perceptual system might simply become insensitive to the amount of correlation with increasing temporal rate.

### Synchrony detection

Like correspondence detection, multisensory perception of synchrony is also systematically modulated by the temporal structure of the signals. To investigate the determinants of synchrony detection, Fujisaki and Nishida^5^,^20^ presented periodic sequences of visual and auditory stimuli while parametrically manipulating temporal frequency and phase shifts of the signals^5^ (Experiment 1, Fig. 3c), or their lag and complexity^20^ (Experiment 1, Fig. 3d). The model output 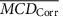 again tightly replicates all empirically determined trends (Fig. 3c,d; see Methods and Supplementary Table 1).

### Temporal order judgment

The temporal order judgment is a classic paradigm to study temporal aspects of multisensory perception. When participants report on the temporal order of simple visual and auditory signals, response probabilities as a function of physical lag usually follow a sigmoidal distribution (often modelled for simplicity as cumulative Gaussian or logistic functions, see, for example, Spence *et al*.^21^, Experiment 2). 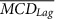 (equation 7) accurately reproduces the same distribution in the commonly measured range around physical synchrony (0ms lag), and displays the distinctive sigmoidal shape of temporal order judgments responses (Fig. 3e and Supplementary Table 1). However, for longer lags beyond the integration window of the MCD, 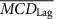 output would drop to 0 as the filtered input signals are not overlapping and thus no order assignment would be possible. Conversely, human performance clearly becomes easier with longer lags. We would argue though that responses at longer lags are based on other cognitive detection mechanisms and not on the MCD, which models temporal processing in the perceptual range^22^.

### Synchrony judgment

Another popular procedure to investigate temporal aspects of multisensory perception is the synchrony judgment task. Participants are typically presented with one visual and one auditory stimulus, and have to report whether such signals appear to be subjectively synchronous or not. When plotted against lag, synchrony judgments usually display asymmetric bell-shaped response distributions, with the perception of synchrony peaking at short lags (for example, see ref. 23). Once again, 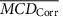 (equation 6) faithfully replicates human performance^23^ (Experiment 1, see Fig. 3f, and Supplementary Table 1), and it displays, due to differences in the filtering of the two input signals, the characteristic asymmetry often found in synchrony judgments. That is, the MCD model can explain the standard shape of synchrony judgments solely based on the response of a simple correlation detector (for example, without the need to assume multiple decision criteria; see ref. 24).

All in all, these simulations show that the MCD can reproduce human multisensory temporal processing under a broad range of experimental manipulations.

### MCD and optimal cue integration

Over the past decade, multisensory integration has been predominantly modelled in terms of Bayesian Decision Theory^25^. The main finding is that humans integrate redundant multisensory information in a statistically optimal fashion, thereby maximizing accuracy and precision of combined sensory estimates^26^. Being based on multiplicative interactions, the correlation detector of the MCD is naturally suited to implement Bayes-optimal multisensory integration.

**Figure 4:**
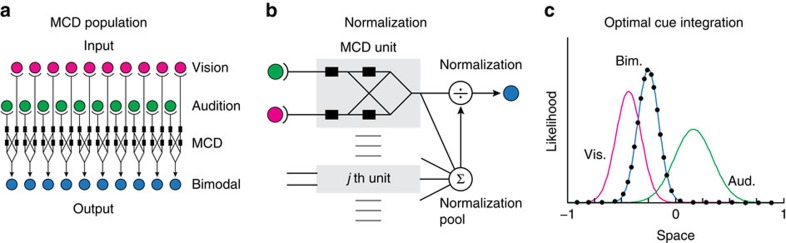
MCD and optimal cue integration. (**a**) A population of spatially tuned MCDs. Each unit receives information from a limited region of visual and auditory space (**b**) normalization. The output of each unit (*MCD*_Corr_, equation 4) is normalized across units to get a probability distribution of model response over space. (**c**) MCD and optimal cue integration. Normalized responses of a population of MCDs tuned to a preferred stimulus dimension (for example, space) to visual and auditory stimuli with a spatial offset. The green and magenta lines represent MCD responses to spatially offset unimodal visual and auditory stimuli, respectively. The blue line represents the response of the MCD to the bimodal audiovisual stimuli, while the black dots represent the prediction of Bayes-optimal integration.

To illustrate this in the case of spatial localization of visual and auditory stimuli^27^, we simulated a population of MCDs, each receiving inputs from spatially tuned visual and auditory units (Fig. 4a). Each input unit has a receptive field that is Gaussian in shape and with a width that is inversely proportional to the reliability of the input (Supplementary Fig. 6, see refs 26, 28). Thus the reliability of a signal's estimate is the emergent property of neuronal tuning to a particular stimulus, which changes with the type of stimuli used, and neural noise. The output of each MCD unit (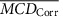, equation 6) is then normalized by dividing it by the sum of the responses of all units. This divisive normalization, which is biologically plausible^29^,^30^, eventually provides an estimate of the probability distribution of stimulus location (see, Fig. 4b).

To better highlight the weighting behaviour resulting from optimal multisensory integration, we introduced a small offset between the spatial locations of the visual and auditory stimuli, and rendered the auditory input less reliable than vision (wider receptive fields). Stimuli consisted of impulses lasting for one sample (sampling frequency 1 kHz), which were embedded into temporal sequences of samples with constant, near-zero values. The model output was time-averaged over a 2 s window. Figure 4c shows the output of the model for unimodal and bimodal signals (continuous lines), and the prediction of optimal cue integration (that is, the normalized product of the unimodal distributions, represented by the dots^25^). The combined response predicted by the MCD matches the joint probability distribution on which the optimal percept is based (see Supplementary Note 3, for a description of how this population model can be extended to include weaker forms of coupling across the signals, represent priors, and implement spatial recalibration).

Despite the success of Bayesian models in predicting multisensory integration, it is still unclear how they can account for the breakdown of optimal integration in the presence of temporal conflicts, that is when the signals are not synchronous. Given that *MCD*_Corr_ responds maximally to synchronous and correlated stimuli, this population model can naturally account for the temporal constraints of optimal integration, as with longer lags the filtered input signals are non-overlapping, and thus no integration would be possible. Hence, going beyond earlier neural models of multisensory integration^31^,^32^, this model directly deals with time-varying signals, and it can jointly account for both optimal cue integration and for its breakdown when temporal conflicts occur^3^.

## Discussion

Overall, these results strongly suggest that the MCD may represent the elementary unit for multisensory processing: individual units solve the correspondence problem, by detecting correlation, lags and synchrony across the senses, and integrate only those signals that are likely causally related; larger populations of MCDs perform optimal multisensory integration. So far, these diverse phenomena have only been partially explained with separate *ad hoc* models^5^,^24^,^25^,^26^,^28^,^29^,^33^, or have never been explained at all (like the detection of temporal order across the senses). The MCD parsimoniously captures all such perceptual challenges, and provides a common explanation to both the spatial and the temporal aspects of multisensory integration with a simple neural architecture whose biological plausibility is supported by a vast literature on the physiology of motion perception^14^ and stereoscopic vision^11^. What is more, the MCD might help bridging the gap between physiology and behaviour, as it can also be used to model the responses of multimodal neurons, just like the Hassenstein–Reichardt detector has been used as a model for motion-sensitive neurons^14^.

Although correlation detection provides obvious behavioural benefits such as solving the correspondence problem and integrating related signals, it is not equally clear why the nervous system should have a dedicated detector to constantly monitor lags across the senses. Multisensory signals, however, often reach our senses with some relative lags (e.g., due to differences in the generation process of the signals in each modality, the speed of propagation, which is slower for sound, or the neural latencies during transduction and transmission). Hence, the nervous system must quickly detect and actively compensate for such delays (i.e., temporal recalibration, see refs 34, 35). In this context, a lag detector would provide the necessary information to drive temporal recalibration and restore perceptual synchrony. Given the analogies between motion perception and multisensory temporal processing, it would be reasonable to hypothesize that the same mechanisms underlying visual motion adaptation^36^ might also serve multisensory temporal recalibration^19^,^34^ (Supplementary Fig. 8).

Correlation detection has often been advocated as a universal computational mechanism, which simultaneously operates in multiple sensory systems (for example, visual motion, binocular disparity and binaural hearing) throughout the animal kingdom^37^, including mammals, avians and even invertebrates. The present study suggests that the brain parsimoniously implements analogous principles of sensory processing also to combine signals across the senses, not just within. Because of its biologically inspired nature and its effectiveness in predicting psychophysical results, the MCD provides a unified general theory of multisensory processing—one that is capable of generating quantitative predictions at many different levels, from neurons^14^,^38^ to behaviour^32^.

## Methods

### Model

The MCD model consists of a first filtering stage, where time-varying visual and auditory signals (*S*_V_(*t*), *S*_A_(*t*)) are independently low-pass filtered, and a subsequent integration stage, where the two signals are combined through linear operations (multiplication or subtraction). Low-pass filters (*f*) were modelled as exponential functions of the form (cf. Burr *et al*.^6^):


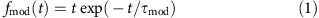


*τ*_*mod*_ is the modality-dependent temporal constant of the filter. On the basis of the empirical results, we estimated these constants to be *τ*_V_=87 ms and *τ*_A_=68 ms for the visual and auditory filters, respectively. The second filter, which for simplicity we assumed to be identical in both subunits of the detector, was estimated as *τ*_AV_=786ms (cf. fitting details below).

Each subunit (*u*_1_, *u*_2_) of the detector independently combines multisensory information by multiplying the filtered visual and auditory signals as follows:


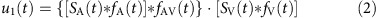



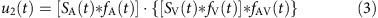


To this end, the signals are convolved (*) with the low-pass temporal filters. The response of the subunits are eventually multiplied or subtracted.


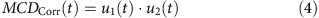



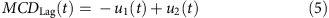


The resulting time-varying responses represent the local temporal correlation (*MCD*_Corr_) and lag (*MCD*_Lag_) across the signals (Supplementary Fig. 1). To reduce such time-varying responses into a single summary variable representing the amount of evidence from each trial, we simply averaged the output of the detectors over a window of 3 s—three times the duration of each trial:


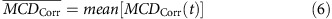



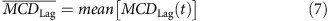


A Matlab implementation of the MCD model is provided in Supplementary Software 1. Given that we were especially interested in how close the model could reproduce the shape of the empirical classification images, the temporal constants of the model (that is, *τ*_V_, *τ*_A_ and *τ*_VA_) were free parameters that we fitted to maximize the correlation between predicted and empirical classification images of the averaged observer (see reverse-correlation analyses). The fitting was based on an optimization algorithm (fminsearch, Matlab) that maximizes the similarity (Pearson's correlation) between empirical and predicted classification images. To calculate the classification images from MCD responses, we ranked the responses of the model across trials, and divided them into two classes (one for each response category) with the same relative frequency that we determined empirically. For example, if participants classified 40% of the trials as ‘common cause', the 40% of trials with highest 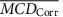 outputs were classified as ‘common cause'—the remaining trials as ‘different causes'.

The main difference between empirical and predicted classification images is that overall the predicted ones have higher amplitudes. This is an expected finding, given that in the model we did not consider the detrimental effects of noise, which would naturally arise at any stages of the detector, including late noise occurring at the decision stage, and which would be additive to the detector's outputs 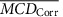 and 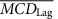. Since noise is unlikely to be correlated across the senses, it would only reduce the cross-correlation between the streams of sensory information, and corrupt perceptual decision-making, hence reducing the overall amplitude of the empirical classification images. Note that the predicted classification images in Fig. 2d–f and Supplementary Fig. 2A–C are vertically scaled for graphical clarity in order to better highlight the similarity in shape of the predicted and empirical results.

To demonstrate how model output can systematically predict human responses, we partitioned all trials into 30 bins (315 responses for each bin, 63 per participant) based on the output of the model (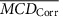 for the causality judgment, and 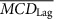 for the temporal order judgment task). For each bin, we calculated the average response of the model and plotted it against the mean response of our participants in these same trials (Fig. 2e,g and Supplementary Fig. 2b–d), while the monotonicity of the relationship between model and human responses was assessed using Spearman correlation.

### Psychophysical task

The experiment consisted of a force-choice dual task, whereby on each trial a train of five impulses was presented to the visual and auditory modalities. The stimuli consisted of sequences of five visual and five auditory impulses randomly presented over an interval of 1 s (average temporal rate 5 Hz, Fig. 2b). Each impulse consisted of a single sample with a value of 1 in an array of samples with a value of 0 (sampling frequency 44.1 kHz). Participants (four naive and C.V.P., age range 22–35 years, one female) observed the stimuli and had to report (1) whether or not the visual and auditory sequences appeared to be causally related and formed perceptual unity (causality judgment, also known as ‘relatedness' task^17^), and (2) the relative temporal order of the two sequences (temporal order judgment). To instruct participants on what we meant by ‘causally related', we told them to imagine that clicks and flashes were all little explosions, and their task was to tell whether the same underlying sequence of blasts caused the clicks and the flashes, or whether light and sound were generated by independent generative processes. Given that we collected a large number of trials (overall *n*=9,450), and that we performed the analyses both at the group level and at the level of the individual participants, a pool of five participants is large enough to provide reliable estimates of the effects under study. To assess whether the dual-task paradigm could qualitatively alter observers' responses, we also performed an additional control experiment in which the causality judgment and temporal order judgment tasks were performed in separate sessions (see Supplementary Note 4 and Supplementary Fig. 9). Overall the results from both experiments were in good agreement with those of the dual-task paradigm.

The experiment was performed in a dark anechoic chamber. Visual stimuli consisted of a white disk (rad=6.5°) of sound-transparent fabric backlit by a white-light-emitting diode. Auditory stimuli were presented from the same location as the visual stimuli by means of a small loudspeaker hidden behind the sound-transparent screen. Both the light-emitting diode and the loudspeaker were operated via a computer soundcard to ensure near-perfect timing of the multisensory signals. The experiment was controlled by custom-built software based on the Psychtoolbox^39^.

The experiment was self-paced, and participants had to press a key to initiate each trial. The temporal structures of the signals were identical across participants. After stimulus presentation, participants responded by pressing one of four keys arranged in a 2 × 2 matrix. The vertical axis of this arrangement represented the response to the causality judgment (top=single cause, bottom=different causes), while the horizontal axis represented the response to the temporal order judgment (left=vision first and right=audition first). Participants were instructed to give an answer even if unsure, taking their time and guessing if necessary. The experiment took place in four sessions of ∼2 h each (including breaks). Every 45 trials, there was a break and a dim table-light was smoothly turned on. Participants were allowed to rest for as long as they wanted, and to minimize boredom they were entertained with a booklet of short jokes. Participants had to press a key to restart the experiment, after which the table-light smoothly turned off. Throughout the experiment, participants' head was constrained with a chin- and a head-rest. The experiment was conducted in accordance to the Declatation of Helsinki and was approved by the Ethics committee of the University of Bielefeld. Participants received 6 Euro per hour, and they provided written informed consent before participating to the experiment.

### Reverse-correlation analyses

To calculate visual classification images, we first sorted the stimuli presented in the experiment according to participants' (or model's, c.f. Modelling) classification responses (single cause vs. multiple causes in the causality judgment, CJ; light vs. sound lead in the temporal order judgment, Fig. 2c; see ref. 40). For each class we calculated the mean cross-correlation across the visual and auditory signals (*S*_V_(*t*)*S*_A_(*t*)) and combined them to obtain the classification images for audiovisual correlation (**K**_Corr_) and lag (**K**_Lag_, Fig. 2c) according to:


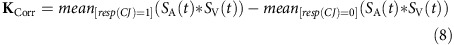



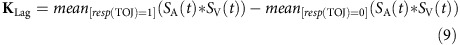


Classification images (**K**_Corr_ and **K**_Lag_) were temporally smoothed by convolution with a temporal low-pass Gaussian filter (*σ*=20 ms). Reverse-correlation analyses were performed individually for each participant (Supplementary Fig. 2), and on the averaged observer (Fig. 2d,f). Given that the stimuli were identical across participants, the response of the averaged observer on each trial was calculated as the mode of the individual responses (that is, if the three out of five participants responded ‘vision first' on a given trial, then the response of the average observer was also ‘vision first').

### Simulation of audiovisual correspondence detection

We simulated visual and auditory signals with statistically the same temporal structures described in Denison *et al*.^17^ (Experiments 1 and 3), and fed them into the MCD (equation 4). Both visual and auditory events consisted of 1ms luminance and loudness impulses.

The MCD response to such signals was transformed into categorical responses in the following way: Given that the behavioural task consisted of a two-alternative forced-choice (that is, participants had to report which of two visual stimuli temporally matched the auditory stimulus^17^), we assumed the underlying decision variable to be the ratio between the 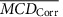 (equation 6) response to the two visual stimuli (match/non-match). That is, we divided the 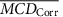 response to the matching stimulus by the response of the model to the non-matching stimulus. Such a variable was then transformed into the proportion of correct responses via a general linear model with a probit link function (assuming additive Gaussian noise). Linear coefficients were fitted over the whole data set (proportion of correct responses, which we computed from the original *d*' measures) included in Fig. 3a,b.

Given that Denison *et al*.^17^ used random temporal structures that changed across trials, we could not faithfully reproduce the exact stimuli used in the original experiment. Therefore for each data-point, we simulated 500,000 random temporal structures for the auditory stimuli, other 500,000 for the matching visual stimuli, and the same amount for non-matching visual stimuli. The decision variable was eventually calculated as the ratio of the median MCD response to the two visual stimuli (that is, median 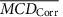 response to the matching visual stimuli divided by the median response to the non-matching visual stimuli).

### Simulation of synchrony detection

The experimental task used by Fujisaki and Nishida^5^,^20^ consisted of a two-alternative forced-choice task, whereby participants reported on the perceived synchrony of auditory and visual stimuli. Assuming synchrony detection to rely on the same mechanisms of correspondence detection, we used MCD to simulate the results of Fujisaki and Nishida^5^ (Experiment 1, see Fig. 3c) and Fujisaki and Nishida^20^ (Experiment 1, see Fig. 3d). This was done by generating stimuli as described in Fujisaki and Nishida^5^ (Experiment 1) and Fujisaki and Nishida^20^ (Experiment 1), and calculating 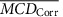 (equation 6). As in the previous simulation, we carefully replicated the temporal structures of the stimuli. However, for simplicity the exact visual and auditory events were replaced by impulses. We used standard procedures to transform the responses of the model into binary responses. This was done by assuming that the response on each trial depended on the ratio between the model's responses for the asynchronous signal, to the model's response for the synchronous signal. Such a variable was then transformed into the proportion of correct responses via a general linear model with a probit link function (assuming additive Gaussian noise). Linear coefficients were fitted over the whole data set included in Fig. 3c,d.

### Simulation of temporal order judgment

To investigate whether 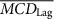 (Equation 7) could reproduce the typical shape of the response distribution of audiovisual temporal order judgments, we fed a pair of visual and auditory impulses with the same lags as in Spence *et al*.^21^ (Experiment 2) into the model. Again, 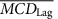 was related to response probabilities using a general linear model with a probit link function (Fig. 2e). Linear coefficients were fitted over the empirical data from Spence *et al*.^21^ (Experiment 2).

### Simulation of synchrony judgment

To see whether 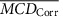 could replicate the response distribution of synchrony judgment tasks, we generated stimuli as in Slutsky and Recanzone^23^ and calculated model responses (Fig. 3f). The exact stimuli were replaced by impulses, but the temporal structures in our simulations were the same as in the original study. 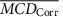 output (equation 6) was related to response probabilities using a general linear model with a probit link function. Linear coefficients were fitted over the empirical data in Fig. 3f (ref. 23) (Experiment 1, 1 kHz condition).

To assess how well the MCD model could predict previous results, we calculated the Pearson correlation and the coefficient of determination between the empirical and the predicted responses (Supplementary Table 1). To test the statistical significance of a linear regression relationship between empirical and predicted responses, we used the F-test (Supplementary Table 1). The F-test requires the residuals of the linear regression to be normally distributed: the validity of this assumption was corroborated for all tests using a Lilliefors test for normality. Regression analyses were performed separately for each simulated study.

## Additional information

**How to cite this article:** Parise, C. V. *et al*. Correlation detection as a general mechanism for multisensory integration. *Nat. Commun.* 7:11543 doi: 10.1038/ncomms11543 (2016).

## Supplementary Material

Supplementary Information Supplementary Figures 1-9, Supplementary Table 1, Supplementary Notes 1-4 and Supplementary References

Supplementary Movie 1 MCD model. The top-part of the Movie represents the MCD model. The luminance of the input and the output units represents the strength of the input and the output, respectively. The bottom-left part of the Movie represents the input (*S_V_*(*t*), *S_A_*(*t*)) and the output (*MCD_Corr_*(*t*) and *MCD_Lag_*(*t*, Equations 4-5) signals. The moving bar represents time. As the bar scans through the output signals, the area under the curves (which changes color) determines the time-average response of the model (dynamically represented by the moving cursor in the bottom-right panel). The bottom-right panel represents the time-averaged impulse response functions (*MCD_Corr_*, *MCD_Lag_* and, Equations 6-7). In essence, this Movie is the dynamic version of Figure 1B and Supplementary Figure 1.

Supplementary Movie 2 Auditory and visual stimuli. The first three visual and auditory stimuli correspond to the three stimuli represented in Figure 2A. Note that in the actual experiment did not use a computer screen, but a white, sound-transparent fabric disk back-lit by a white LED. Auditory stimuli were delivered by a speaker placed behind the fabric disc, next to the LED.

Supplementary Software 1 Matlab implementation of the MCD model. See comments for instructions.

## References

[b1] Stein B. E. (Ed.) The New Handbook of Multisensory Processing MIT Press (2012).

[b2] Ernst M. O. & Bülthoff H. H. Merging the senses into a robust percept. Trends Cogn. Sci. 8, 162–169 (2004). 15050512 10.1016/j.tics.2004.02.002

[b3] Parise C. V., Spence C. & Ernst M. O. When correlation implies causation in multisensory integration. Curr. Biol. 22, 46–49 (2012). 22177899 10.1016/j.cub.2011.11.039

[b4] Parise C. V., Harrar V., Ernst M. O. & Spence C. Cross-correlation between auditory and visual signals promotes multisensory integration. Multisens. Res. 26, 307–316 (2013). 23964482 10.1163/22134808-00002417

[b5] Fujisaki W. & Nishida S. Temporal frequency characteristics of synchrony-asynchrony discrimination of audio-visual signals. Exp. Brain Res. 166, 455–464 (2005). 16032402 10.1007/s00221-005-2385-8

[b6] Burr D., Silva O., Cicchini G. M., Banks M. S. & Morrone M. C. Temporal mechanisms of multimodal binding. Proc. R. Soc. Lond. B Biol. Sci. 276, 1761–1769 (2009). 10.1098/rspb.2008.1899 PMC2674495 19324779

[b7] Spence C. Just how important is spatial coincidence to multisensory integration? Evaluating the spatial rule. Ann. N Y Acad. Sci. 1296, 31–49 (2013). 23710729 10.1111/nyas.12121

[b8] Chen L. & Vroomen J. Intersensory binding across space and time: a tutorial review. Attent. Percept. Psychophys. 75, 790–811 (2013). 10.3758/s13414-013-0475-4 23709064

[b9] Raposo D., Sheppard J. P., Schrater P. R. & Churchland A. K. Multisensory decision-making in rats and humans. J. Neurosci. 32, 3726–3735 (2012). 22423093 10.1523/JNEUROSCI.4998-11.2012 PMC3335889

[b10] Jeffress L. A. A place theory of sound localization. J. Comparat. Physiol. Psychol. 41, 35 (1948). 10.1037/h0061495 18904764

[b11] Ohzawa I. Mechanisms of stereoscopic vision: the disparity energy model. Curr. Opin. Neurobiol. 8, 509–515 (1998). 9751654 10.1016/s0959-4388(98)80039-1

[b12] Hassenstein V. & Reichardt W. System theoretical analysis of time, sequence and sign analysis of the motion perception of the snout-beetle *Chlorophanus*. Z. Naturforsch. 11, 513–524 (1956).

[b13] Adelson E. H. & Bergen J. R. Spatiotemporal energy models for the perception of motion. J. Opt. Soc. Am. A 2, 284–299 (1985). 3973762 10.1364/josaa.2.000284

[b14] Borst A. & Euler T. Seeing things in motion: models, circuits, and mechanisms. Neuron 71, 974–994 (2011). 21943597 10.1016/j.neuron.2011.08.031

[b15] Behnia R., Clark D. A., Carter A. G., Clandinin T. R. & Desplan C. Processing properties of ON and OFF pathways for *Drosophila* motion detection. Nature 512, 427–430 (2014). 25043016 10.1038/nature13427 PMC4243710

[b16] Takemura S.-y. . A visual motion detection circuit suggested by *Drosophila* connectomics. Nature 500, 175–181 (2013). 23925240 10.1038/nature12450 PMC3799980

[b17] Denison R. N., Driver J. & Ruff C. C. Temporal structure and complexity affect audio-visual correspondence detection. Front. Psychol. 3, 1–12 (2012). 23346067 10.3389/fpsyg.2012.00619 PMC3550803

[b18] Sternberg S. & Knoll R. L. in Attention and performance IV ed Kornblum S. 629–85Academic Press (1973).

[b19] Cai M., Stetson C. & Eagleman D. M. A neural model for temporal order judgments and their active recalibration: a common mechanism for space and time? Front. Psychol. 3, (2012). 10.3389/fpsyg.2012.00470 PMC3487422 23130010

[b20] Fujisaki W. & Nishida S. Feature-based processing of audio-visual synchrony perception revealed by random pulse trains. Vision Res. 47, 1075–1093 (2007). 17350068 10.1016/j.visres.2007.01.021

[b21] Spence C., Baddeley R., Zampini M., James R. & Shore D. I. Multisensory temporal order judgments: when two locations are better than one. Percept. Psychophys. 65, 318–328 (2003). 12713247 10.3758/bf03194803

[b22] Lewis P. A. & Miall R. C. Distinct systems for automatic and cognitively controlled time measurement: evidence from neuroimaging. Curr. Opin. Neurobiol. 13, 250–255 (2003). 12744981 10.1016/s0959-4388(03)00036-9

[b23] Slutsky D. A. & Recanzone G. H. Temporal and spatial dependency of the ventriloquism effect. Neuroreport 12, 7–10 (2001). 11201094 10.1097/00001756-200101220-00009

[b24] Yarrow K., Jahn N., Durant S. & Arnold D. H. Shifts of criteria or neural timing? The assumptions underlying timing perception studies. Conscious. Cogn. 20, 1518–1531 (2011). 21807537 10.1016/j.concog.2011.07.003

[b25] van Dam L. C. J., Parise C. V. & Ernst M. O. in Sensory Integration and the Unity of Consciousness eds Bennett David, Christopher Hill 209–229MIT press (2014).

[b26] Ernst M. O. & Banks M. S. Humans integrate visual and haptic information in a statistically optimal fashion. Nature 415, 429–433 (2002). 11807554 10.1038/415429a

[b27] Alais D. & Burr D. The ventriloquist effect results from near-optimal bimodal integration. Curr. Biol. 14, 257–262 (2004). 14761661 10.1016/j.cub.2004.01.029

[b28] Knill D. C. & Pouget A. The Bayesian brain: the role of uncertainty in neural coding and computation. Trends Neurosci. 27, 712–719 (2004). 15541511 10.1016/j.tins.2004.10.007

[b29] Ohshiro T., Angelaki D. E. & DeAngelis G. C. A normalization model of multisensory integration. Nat. Neurosci. 14, 775–782 (2011). 21552274 10.1038/nn.2815 PMC3102778

[b30] Carandini M. & Heeger D. J. Normalization as a canonical neural computation. Nat. Rev. Neurosci. 13, 51–62 (2012). 22108672 10.1038/nrn3136 PMC3273486

[b31] Ma W. J., Beck J. M., Latham P. E. & Pouget A. Bayesian inference with probabilistic population codes. Nat. Neurosci. 9, 1432–1438 (2006). 17057707 10.1038/nn1790

[b32] Fetsch C. R., DeAngelis G. C. & Angelaki D. E. Bridging the gap between theories of sensory cue integration and the physiology of multisensory neurons. Nat. Rev. Neurosci. 14, 429–442 (2013). 23686172 10.1038/nrn3503 PMC3820118

[b33] Shams L. & Beierholm U. R. Causal inference in perception. Trends Cogn. Sci. 14, 425–432 (2010). 20705502 10.1016/j.tics.2010.07.001

[b34] Fujisaki W., Shimojo S., Kashino M. & Nishida S. Recalibration of audiovisual simultaneity. Nat. Neurosci. 7, 773–778 (2004). 15195098 10.1038/nn1268

[b35] Van der Burg E., Alais D. & Cass J. Rapid recalibration to audiovisual asynchrony. J. Neurosci. 33, 14633–14637 (2013). 24027264 10.1523/JNEUROSCI.1182-13.2013 PMC6705173

[b36] Clifford C. W. G. & Langley K. A model of temporal adaptation in fly motion vision. Vision Res. 36, 2595–2608 (1996). 8917820 10.1016/0042-6989(95)00301-0

[b37] Konishi M. in Cold Spring Harbor Symposia on Quantitative Biology, Vol. 55, 575–584Cold Spring Harbor Laboratory Press (1990). 2132838 10.1101/sqb.1990.055.01.055

[b38] Stein B. E. & Stanford T. R. Multisensory integration: current issues from the perspective of the single neuron. Nat. Rev. Neurosci. 9, 255–266 (2008). 18354398 10.1038/nrn2331

[b39] Kleiner M., Brainard D. & Pelli D. What's new in Psychtoolbox-3. Perception 36, 1–16 (2007).

[b40] Knoblauch K. & Maloney L. in Modeling Psychophysical Data in R eds Knoblauch K, Maloney L Ch. 6, 167/194 Springer (2012).

