# Supplementary material for: Correlation detection as a general mechanism for multisensory integration
Source: Nat Commun. 2016 Jun 6;7:11543. doi: 10.1038/ncomms11543 (PMC4897755; doi:10.1038/ncomms11543)
Supplement: Supplementary Information — Supplementary Figures 1-9, Supplementary Table 1, Supplementary Notes 1-4 and Supplementary References [file ncomms11543-s1.pdf]

## Supplementary Figures

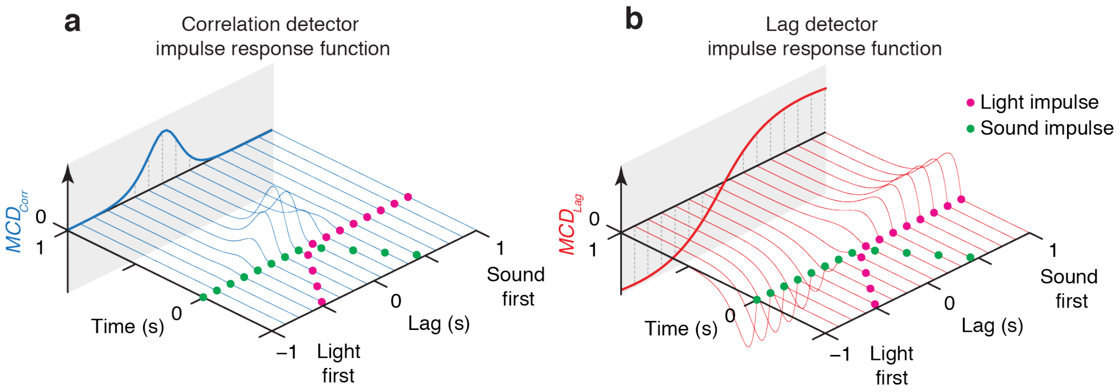


**Supplementary Figure 1 | Impulse response function of the model at various lags.** Impulse response functions (thin lines) represent the output of the model (over time) in response to visual and auditory impulses (magenta and green dots, respectively) as a function of the relative lags across the senses. Panel A represents the output of the correlation detector ($MCD_{Corr}$, Equation 4, Movie 1, bottom-left blue lines), panel B represents the output of the lag detector ($MCD_{Lag}$, Equation 5, Movie 1, bottom-left red lines). Note how the impulse response functions of $MCD_{Corr}$ is maximal at low lags, while the sign of the $MCD_{Lag}$ depends on the relative temporal order of the visual and auditory impulses. Time zero indicates the temporal onset of the last of the two impulses (i.e., either the visual or the auditory impulse, depending on the sign of the lag). The solid continuous lines projected against the vertical plane represent the time-averaged response of the model ($\overline{MCD_{Corr}}$, Equation 6, panel A; $\overline{MCD_{Lag}}$, Equation 7, panel B; see also Figure 1B and Movie 1, bottom-right plot). Such time-averaged responses are vertically scaled for graphical clarity.

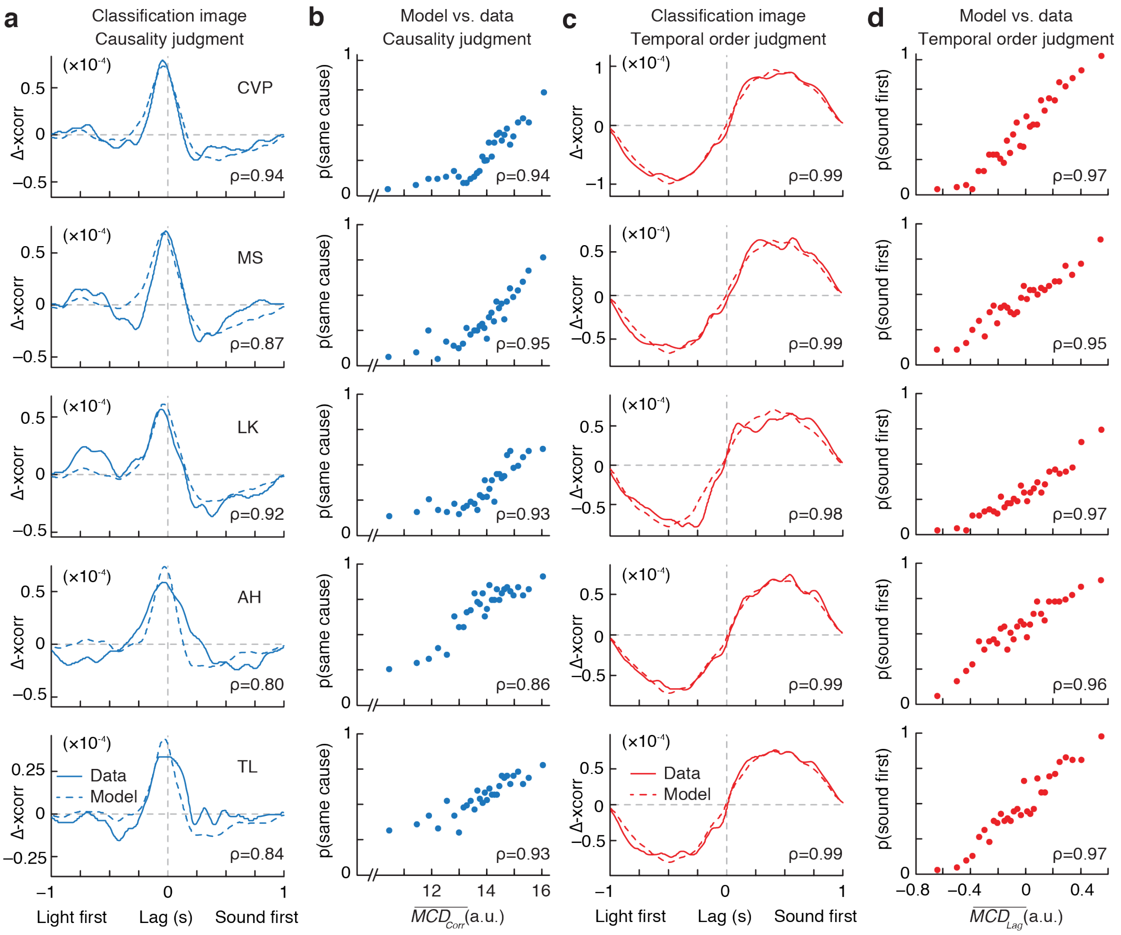


**Supplementary Figure 2 | Individual participants' data.** Each row corresponds to a different participant; the first row represents the data from the author CVP. (A,C) Classification images for the causality and the temporal order judgments, respectively. Solid lines represent the empirical classification images (averaged across observers), dashed line represent the one predicted from the MCD model using the same stimuli. Positive values on the y-axes represent positive association to "same cause" and "sound first" responses, respectively. The number at the bottom-right of each panel represents the Pearson correlation between empirical and predicted classification images. Predicted classification images are vertically scaled. (B,D) Model output (Equations 6-7) plotted against human responses. Each dot corresponds to 63 responses. Note that for these plots, model predictions were based on the temporal constants of the MCD fitted on the averaged observer. The number at the bottom-right of each panel represents the Spearman correlation between model output and human responses.

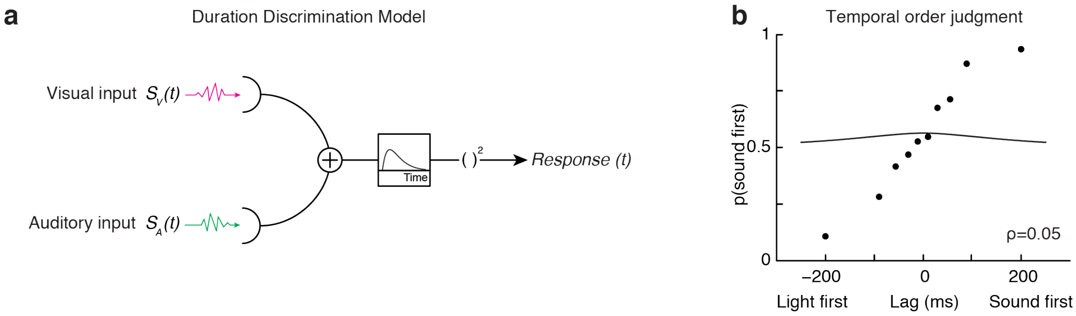


**Supplementary Figure 3 | Duration Discrimination Model (DDM). A.** This model combines multisensory inputs with a sum. Panel **B** shows that the DDM cannot perform temporal order judgments (compare to Figure 3E; data taken from[1]). ρ indicates the correlation between empirical and predicted performance.

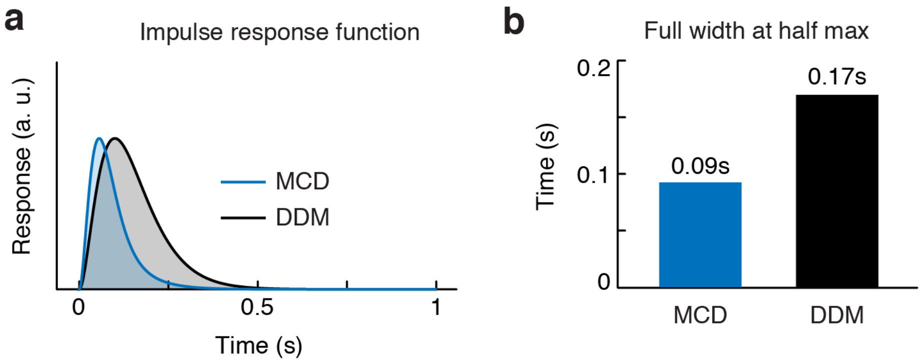


**Supplementary Figure 4 | Impulse response functions**. **A.** Impulse response function of the MCD model (blue) and the DDM (black). **B.** Width (i.e., temporal resolution) of the impulse response functions in Panel A measured in terms of full width at half-height. Although our model has longer temporal constants than the DDM, it displays a nearly two-fold sharper impulse response function.

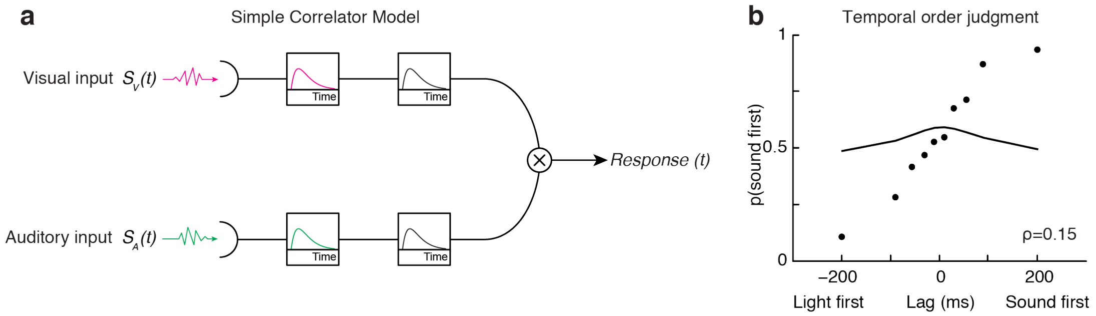


**Supplementary Figure 5 | Simple Correlator Model (SCM). A.** This model is a simplified version of the MCD model obtained by removing the cross-wired sub-units and the opponent stage. Note the similarity with the conceptual model of Fujisaki and Nishida[2]. Given the absence of these components, the SCM cannot detect the relative temporal order of the signals (Panel **B**; compare with Figure 3E). ρ indicates the correlation between empirical and predicted performance.

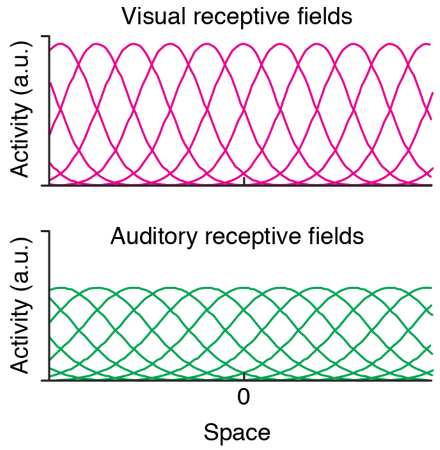


**Supplementary Figure 6 | Spatial receptive fields of the input units.** Receptive fields are modeled as Gaussian tuning functions. The width of the tuning function determines the spatial resolution, which is lower for audition (wider tuning) than for vision (narrower tuning). The MCD units in Figure 4A and Supplementary Figure 8A receive input only from spatially aligned visual and auditory receptive fields.

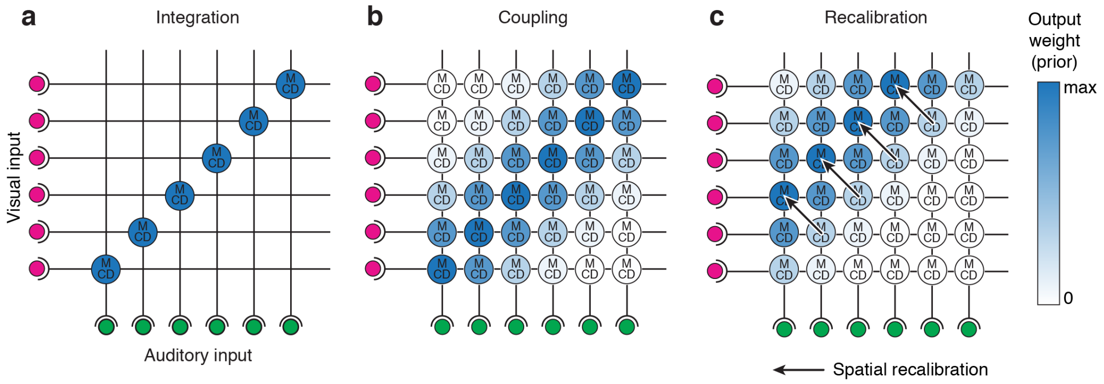


**Supplementary Figure 7 | Multisensory spatial interactions**. **(A)** Spatial integration (mandatory fusion). Corresponds to situation shown in Figure 4A, redrawn in a different arrangement. **(B)** Weaker form of spatial coupling between the input signals. This is achieved by a gradient of output weights (blue) to the matrix of MCD units that are placed at the joints of the visual and auditory units. **(C)** Spatial recalibration is achieved by updating the output weights, such that the mapping between the multisensory inputs is shifted (arrows) relative to the situation shown in panel B.

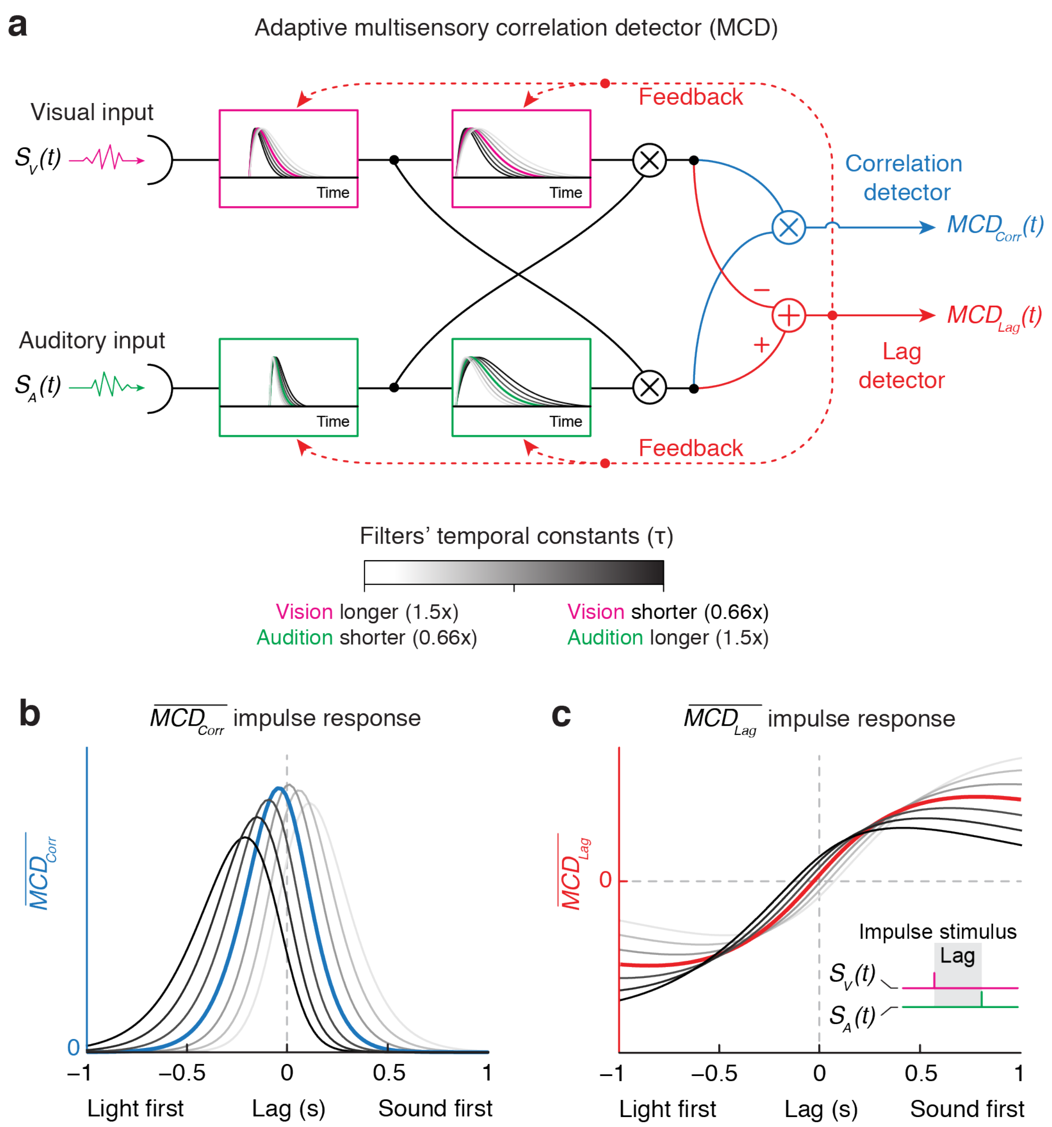


**Supplementary Figure 8 | Implementation of an adaptive MCD that can perform temporal recalibration. (A)** If the output of the lag detector ($MCD_{Lag}$, Equation 5) of the adaptive multisensory correlation detector were used in a feedback loop to differentially modulate the temporal constants ($\tau$) of the visual (top) and auditory (bottom) low-pass temporal filters (Equation 1), the MCD would dynamically adapt to lags across the senses. **(B)** To illustrate how differentially changing the temporal constants of visual and auditory signals could be used to shift perceptual simultaneity in response to adaptation lags, we calculated the (time-averaged) impulse response functions of the model ($\overline{MCD_{Corr}}$, Equation 6, panel B; $\overline{MCD_{Lag}}$, Equation 7, panel C) at various lags. For this, we applied a gain factor to the temporal constants in one modality (e.g., vision) and its inverse to the other modality (e.g. audition). Different modulations of the temporal constants of the filter (panel A), and their outcomes (panels B,C), are represented using different shades of gray. Note how the temporal constants of the visual filters (panel A, top) get wider from black to white (delayed temporal response), while for the auditory filters they get narrower (brisker temporal response; panel A, bottom). Magenta and green temporal filters in panel a represent the shape of the filters fitted from the psychophysical experiment and correspond to the filters in Figure1A. Changes in the temporal constants of the filters lead to systematic shifts of the impulse response functions (panels B,C), and hence in the location and shape of the temporal window of perceptual simultaneity. The solid blue and red lines in panels C and C depict the impulse response functions corresponding to the temporal constants fitted from psychophysical data, and represented in Figure 1B.

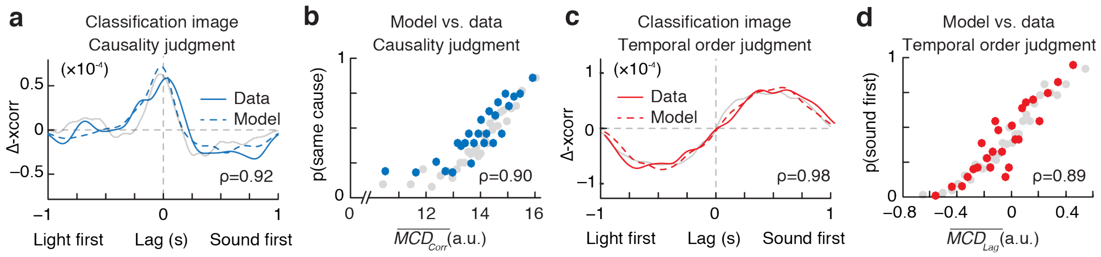


**Supplementary Figure 9 | Single-task data.** Classification images for the causality and the temporal order judgments, Panels **A** and **C**, respectively. Solid lines represent the empirical classification images averaged across observers, dashed lines represent the one predicted from the MCD model using the same stimuli, and gray lines represent the performance in the Dual-task experiment (Figure 2D-F). Positive values on the y-axes represent positive association to "same cause" and "sound first" responses, respectively. Predicted classification images are vertically scaled. Classification images were smoother with a Gaussian kernel (σ=70ms). (**B, D**) Model output (Equations 6-7) plotted against human responses. Compare this figure with Figure 2D-G. ρ indicates the correlation between empirical and predicted performance.

## Supplementary Table

| | Pearson correlation | r-squared | F | Degrees of freedom | p-value |
|---|---|---|---|---|---|
| **Figure 3A-B** | 0.90 | 0.81 | 87.337 | 1, 20 | <0.0001 |
| **Figure 3C-D** | 0.98 | 0.96 | 938.46 | 1, 41 | <0.0001 |
| **Figure 3E** | 0.99 | 0.98 | 505.8 | 1, 8 | <0.0001 |
| **Figure 3F** | 0.99 | 0.99 | 1239.3 | 1, 9 | <0.0001 |

**Supplementary Table 1**
**Statistical analyses of the goodness of fit of the simulations in Figure 3.** Analyses include Pearson correlation, coefficient of determination (r-squared), and the F-test for the linear regression (including degrees of freedom and p-values).

## Supplementary Notes

**Supplementary Note 1. Duration Discrimination Model[3] (DDM).** To account for the discrimination of duration of intervals marked by visual and auditory impulses, Burr and colleagues[3] proposed a simple model that is capable of processing the such impulse signals. Although such a model, hereafter referred to as Duration Discrimination Model (DDM), was devised specifically to account for duration discrimination of temporal intervals–a different aspect of temporal processing that we do not aspire to model–its design could in principle implement some aspects of multisensory synchrony perception. Therefore, we can assess to what extent this model for duration discrimination can account also for the current observations.

In the DDM, multisensory signals are summed, filtered, and squared (Supplementary Figure 3A). The low-pass filter ($f_{AV}(t)$) has a shape determined by Equation 1 with a temporal constant of 100ms. In formal terms, the model can be described by the following equation:

$$Response(t) = \{[S_V(t) + S_A(t)] * f_{VA}(t)\}^2 \quad \text{(Equation 10)}$$

The output of the filter is eventually time-averaged to reduce it to a single value representing the amount of evidence for perceptual judgments.

Given that the DDM sums multisensory signals, it will not be able to implement Bayes optimal multisensory integration, as that requires multiplicative interactions across the senses. What is more, summing audiovisual signals implies that every combination of visual and auditory stimuli that yields the same sum will appear identical for such a model. For example, the DDM cannot tell the difference between a signal consisting of three clicks and one flash, or three flashes and one click. Likewise, it will be insensitive to the relative temporal order of the two signals, even at long separations.

To formally assess this, we used the DDM to simulate human responses as assessed in previous studies (e.g. see Figure 3; simulations were performed using the same logic described in the Simulation section of the Online Methods, but the predictions were based on Equation 10). The DDM proved general enough to replicate several aspects of multisensory synchrony perception (references[3-5]; cf. Figure 3A-D), but not the asymmetry and the leftward shift of synchrony judgments (reference[6]; cf. Figure 3F). However, this model cannot perform temporal order judgments (reference[1]; see Supplementary Figure 3B). All in all, besides discriminating the duration of temporal intervals, the DDM is flexible enough to perform also certain aspects of multisensory synchrony perception. However it cannot detect the relative temporal order across the senses and it cannot implement optimal multisensory integration.

On a side note, one difference between the MCD model and the DDM is that the MCD model needed much longer temporal constants to reproduce human performance (e.g., the temporal constant of the multisensory filter of the DDM was 100ms, while in the MCD model it was 786ms). The difference in the estimated temporal constants of the two models can be easily explained in terms of the different mathematical properties of the two models. Specifically, Burr and colleagues[3] modeled the combination of multisensory signals as a sum of the individual signals, which is later low-pass filtered and squared (see Supplementary Figure 3A); while, in the MCD, multisensory signals are first filtered, and then they are multiplied to one another (Figure 1A). The different integration mechanisms lead to major differences in the temporal resolution of the models, which can be measured by analyzing the impulse response functions (e.g., response of the model to a visual impulse and an auditory one presented at the same time) of the two models. Looking at Supplementary Figure 4A, it can be clearly appreciated that, despite having longer temporal constants, the impulse response function of the MCD model is sharper than the response of the DDM. The difference in temporal resolution of the responses of the two models can be measured in terms of full width at half-height of the impulse response functions (Supplementary Figure 4B): The MCD model has roughly a two-fold faster impulse response than the DDM.

This finding shows another important property of the MCD model: due to the cross-wiring and the multiplicative interactions, it entails only minor losses in resolution. This is a desirable property for any model because high temporal resolution requires neurons signaling at a high bitrate, which in turn involves high metabolic costs[7] (i.e., the cost of a bit, as measured in terms of consumption of ATP, increases with bitrate). Therefore, the neural architecture that is required for biologically implementing a MCD would be highly energy-efficient.

**Supplementary Note 2. Simple Correlator Model.** Fujisaki and Nishida[2] have already proposed that multisensory synchrony detection might rely on cross-correlation operations. However, how such a cross-correlation may be instantiated has never been formally described. In the following simulations we will develop a Simple Correlator Model analogous to the conceptual scheme proposed by Fujisaki and Nishida[2], and assess to what extent such a simpler scheme can account for the empirical findings.

This Single Correlator Model consists of a filtering stage, where time-varying visual and auditory signals ($S_V(t)$, $S_A(t)$) are independently low-pass filtered for two times (Supplementary Figure 5A). The shapes of the filters are described by Equation 1. The first filters ($f_A(t)$ and $f_V(t)$) have a temporal constant that is specific to each modality, whereas in the second filtering stage the filters ($f_{AV}(t)$) have the same constant in both senses. Finally, the two filtered signals are combined through multiplication:

$$Response(t) = \{[S_A(t) * f_A(t)] * f_{AV}(t)\} \cdot \{[S_V(t) * f_V(t)] * f_{AV}(t)\} \quad \text{(Equation 11).}$$

In line with previous simulations, the response of the model was reduced to a single value representing the amount of sensory evidence by averaging the output of the model over time. Note the similarity with the model presented in Figure 1C of Fujisaki and Nishida[2]. Of the three conceptual models described in Figure 1A-C of Fujisaki and Nishida[2], we decided to simulate this one because it has more degrees of freedom (3, like our model), and hence better chances than the other two models to replicate human responses. Thus, this choice represents the most conservative approach.

Although a simple correlator of this kind would be capable of detecting synchrony across the senses, it cannot detect the relative temporal order of the sensory signals. As such this implementation of the model would also not have any information to dynamically recalibrate to temporal offsets between lights and sounds (as they often occur in nature due to the different speed of sound and light). To detect the relative temporal order of arrival of visual and auditory information, the MCD model includes the two sub-units (Equations 2-3) and the opponent stage (Equation 5). Without such additional features, a simple correlator will be inevitably blind to the relative temporal order across the senses. This can easily be demonstrated by simulating the results of Spence et al.[1] (Figure 3E).

Given the analogy between the Simple Correlator Model and the model that has been conceptually proposed before by Fujisaki and Nishida[2] to explain synchrony detection, we used the data from that study (i.e., see Figure 3C-D) to fit the temporal constants of the Simple Correlator Model. With such a constrained model, we simulated the study of Spence et al.[1]. Simulations were performed using the same logic as in previous simulations (see Online Methods, Simulation section). Supplementary Figure 5B clearly shows that a Simple Correlator Model is unable to detect the temporal order of arrival of visual and auditory information. However, such a model can capture other aspects of simultaneity perception, such as the behavioral results of Fujisaki and Nishida[2]

**Supplementary Note 3. Optimal multisensory integration.** So far, we only considered the case of mandatory fusion[8] between visual and auditory information, whereby the brain completely fuses multisensory information so that the combined estimate retains no information about the individual components feeding into the integrated percept. Without such information, the brain would be blind to any possible conflicts across the senses, thus making it impossible to dynamically adapt (i.e., recalibrate) to potential spatial conflicts across the senses[9]. However, this framework can be further extended to

include not only mandatory fusion of multisensory spatial information, but also weaker forms of multisensory spatial coupling–i.e. the continuum between independence of the sensory estimates all the way to mandatory fusion–, which would allow for the breakdown of integration with spatial conflicts, and for spatial recalibration of the visual-auditory estimates (e.g., see[10]).

To illustrate this, it is convenient to redraw the MCD population model from Figure 4A in a slightly different arrangement, making the two-dimensional nature of the estimation problem more transparent (Supplementary Figure 7A). Note, however, that Figure 4A and Supplementary Figure 7A are identical models containing a fixed mapping between the spatially tuned auditory and visual population of neurons. The mapping along the diagonal represents mandatory fusion corresponding to the Bayesian approach as explained in Figure 4. Again, sensory integration occurs only when the inputs arrive approximately simultaneously at the unisensory units, as otherwise the MCDs will not respond.

In order to be able to represent also weaker forms of spatial coupling between the sensory inputs, this mapping has to be weakened. This is done by connecting the matrix of nodes between the visual and auditory units with MCDs (Supplementary Figure 7B). There is a gain factor applied to each MCD such that the gradient of weights in this matrix can take on any shape, and the output of each unit is eventually normalized like in Figure 4B[11]. If the weights are non-zero on the diagonal and 0 everywhere else, this is identical to mandatory fusion as shown in Supplementary Figure 7A. If the weights are equal throughout the entire matrix, the inputs would be left spatially unaltered, corresponding to no spatial interaction between the signals (spatial independence). Consequently, an intermediate situation between spatial independence and mandatory fusion is given by a gradient of weights oriented perpendicular to the diagonal that provides the mapping between the inputs (Supplementary Figure 7B). The steeper this gradient, the higher is the degree of coupling between the inputs.

This form of spatial interaction between multisensory estimates has previously been described by Ernst and Di Luca[12] using a Bayesian modelling approach[13]. Here we illustrate how this form of multisensory interaction can be implemented using MCDs (Equation 4) as basic units for multiplication. In the Bayesian framework, the coupling is provided by a prior representing the spatial co-occurrence statistics between the signals, accordingly termed "Coupling Prior"[13]. That is, a tight spatial correlation between the input signals leads to a well-defined mapping between the signals (Coupling Prior) and hence to more complete fusion, while the absence of such a correlation is equivalent to no mapping between the signals (i.e., equal weights throughout the matrix). This corresponds to a flat prior and independence between the signals (i.e. no spatial interactions). In the neural model proposed here, the prior is represented by the gradient of output weights (indicated by the blue shaded MCD units in Supplementary Figure 7B). Thus, the shape of the gradient determines the way the multisensory signals interact in space. For example, if this gradient becomes flat well off the diagonal without dropping to zero, multisensory signals that activate MCD units away from the diagonal will be treated as independent. Note, that this is nothing else than coding for the breakdown of integration with increasing spatial discrepancy between the inputs[10].

The output weights do not have to be fixed, but should be adaptable just as a Bayesian prior would be. A Bayesian prior becomes updated with changes in the statistical distribution of the inputs that represent the environment. For example, if the mapping between the signals changes (as would happen when wearing distorting lenses), the prior would get adapted to follow this new mapping thereby recalibrating the multisensory processes. In the neural model discussed here, such a spatial recalibration would be represented as shifts of the output weights (Supplementary Figure 7C, black arrows) driven by previous MDC responses. For a more complete discussion on how recalibration can be represented by the Bayesian model, see Ernst and Di Luca[12].

It is further worth noting that this scheme is closely related to the causal inference framework put forward by Körding et al.[14] which can be represented by the combination of a flat prior (P(independent causes)) and a prior along the diagonal (P(common cause)). Thus, MCDs could also form the basic structure for the neural implementation of causal inference.

All in all, this simple network of MCDs simultaneously captures the spatial and temporal attributes of multisensory integration, and it can be easily extended to include also weaker forms of coupling between cues, spatial recalibration, spatial breakdown of integration, the representation of priors and causal inference.

**Supplementary Note 4. Single-task experiment.** During the psychophysical task reported in the main text, participants had to simultaneously perform a causality judgment and a temporal order judgment task. To investigate whether such a dual-task paradigm might introduce systematic biases in the psychophysical judgments, we run a control experiment where participants performed the same psychophysical experiment, but causality and temporal order judgments were performed in different sessions (Single-task).

Five participants (4 naïve and author CVP, age range 20-36 years, 2 females) took part in the experiment. Overall each participant performed 720 trials (360 causality judgments, and 360 temporal order judgments). Compared to the original experiment, the smaller number of trials for each type of judgments (1890 vs. 360), was selected because the single task paradigm is necessarily two-times longer to test. Given that we did not use these data to constrain the parameters of the model (unlike the data from the main study), this smaller sample size is sufficient to assess any meaningful difference between the two experiments.

Within the limits of noise, the data collected in the Single-task does not show systematic deviations from the Dual-task condition (compare Supplementary Figure 9 with Figure 2D-G). More importantly, using the temporal constants fitted in the original experiment, the model could faithfully replicate the observed responses of this Single-task (using the same modeling approach as described in the dual-task experiment). Based on this result we can exclude any meaningful interactions between temporal order judgments and causality judgments tasks.

## Supplementary References:


1 Spence, C., Baddeley, R., Zampini, M., James, R. & Shore, D. I. Multisensory temporal order judgments: when two locations are better than one. *Perception & Psychophysics* **65**, 318-328 (2003).

2 Fujisaki, W. & Nishida, S. Temporal frequency characteristics of synchrony-asynchrony discrimination of audio-visual signals. *Experimental Brain Research* **166**, 455-464 (2005).

3 Burr, D., Silva, O., Cicchini, G. M., Banks, M. S. & Morrone, M. C. Temporal mechanisms of multimodal binding. *Proceedings of the Royal Society of London. Series B, Biological Sciences* **276**, 1761-1769 (2009).

4 Denison, R. N., Driver, J. & Ruff, C. C. Temporal structure and complexity affect audio-visual correspondence detection. *Frontiers in Psychology* **3** (2012).

5 Fujisaki, W. & Nishida, S. Feature-based processing of audio-visual synchrony perception revealed by random pulse trains. *Vision research* **47**, 1075-1093 (2007).

6 Slutsky, D. A. & Recanzone, G. H. Temporal and spatial dependency of the ventriloquism effect. *Neuroreport* **12**, 7-10 (2001).

7 Laughlin, S. B., van Steveninck, R. R. d. R. & Anderson, J. C. The metabolic cost of neural information. *Nature neuroscience* **1**, 36-41 (1998).

8 Hillis, J., Ernst, M. O., Banks, M. & Landy, M. Combining sensory information: Mandatory fusion within, but not between, senses. *Science* **298**, 1627-1630 (2002).

9 Wozny, D. R. & Shams, L. Recalibration of auditory space following milliseconds of cross-modal discrepancy. *The Journal of neuroscience* **31**, 4607 (2011).

10 Ernst, M. O. in *The new handbook of multisensory processes* (ed B.E. Stein) 1084–1124 (MIT Press, 2012).

11 Ohshiro, T., Angelaki, D. E. & DeAngelis, G. C. A normalization model of multisensory integration. *Nature Neuroscience* **14**, 775-782 (2011).

12 Ernst, M. O. & Di Luca, M. in *Sensory cue integration.* (eds J. Trommershäuser, M. Landy, & K. Körding) 224-250 (Oxford University Press, 2011).

13 Ernst, M. O. in *Perception of the human body from the inside out* (eds G Knoblich, IM Thornton, M Grosejan, & M Shiffrar) 105–131 (Oxford University Press, 2005).

14 Körding, K. P. *et al.* Causal inference in multisensory perception. *PLoS ONE* **2**, 943 (2007).
